# Supplementary material for: Comprehensive Annotation and Functional Exploration of MicroRNAs in Lettuce
Source: Front Plant Sci. 2021 Dec 24;12:781836. doi: 10.3389/fpls.2021.781836 (PMC8739914; doi:10.3389/fpls.2021.781836)
Supplement: Supplementary file 3 [file Data_Sheet_9.ZIP › GSM2230751.plot/Lsa-miRN1690_Lsat_1_v5_gn_4_145880.1_62_TPlot.pdf]

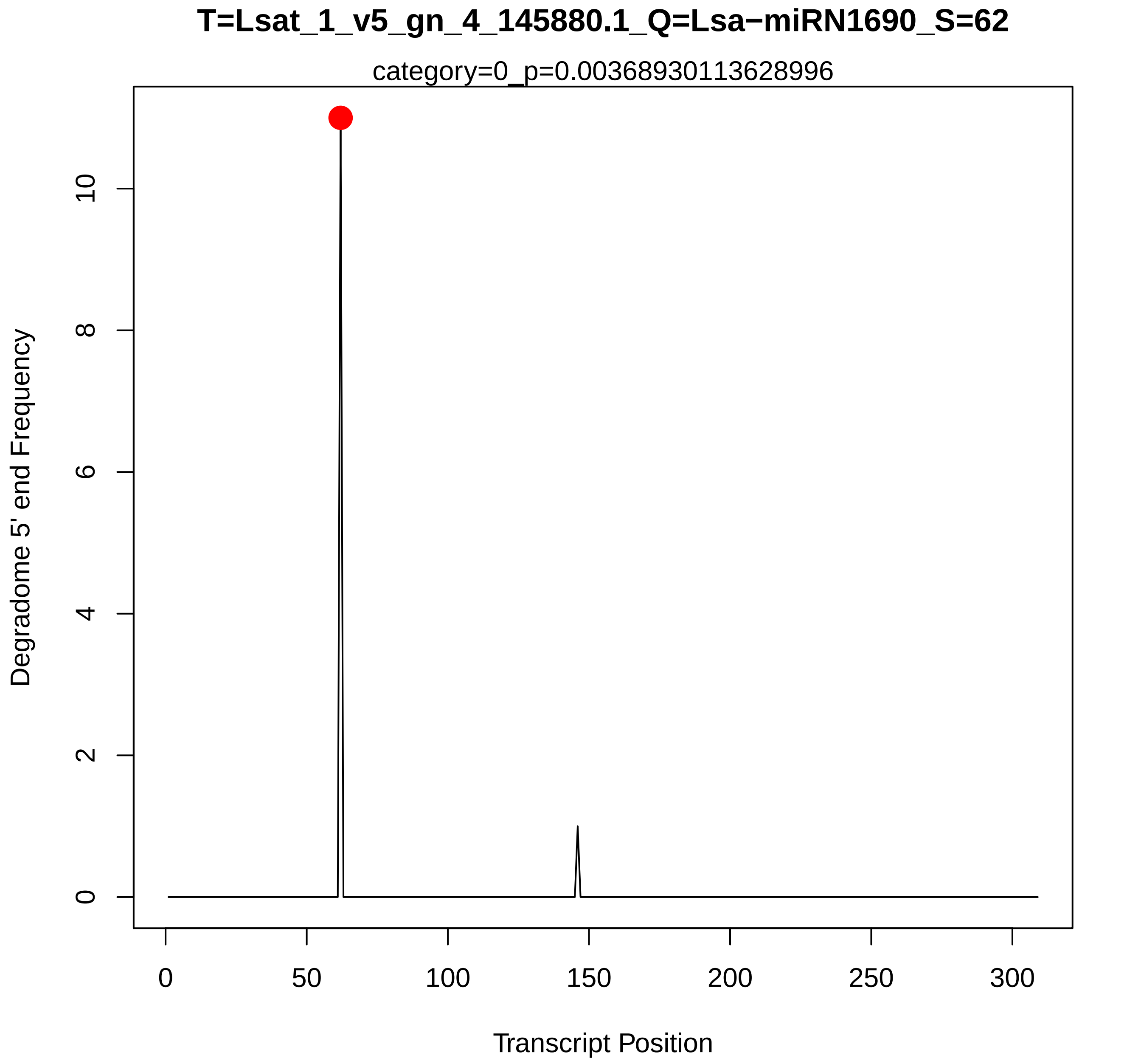

T=Lsat_1_v5_gn_4_145880.1_Q=Lsa−miRN1690_S=62
category=0_p=0.00368930113628996
Degradome 5' end Frequency
0
2
4
6
8
10
0
50
100
150
200
250
300
Transcript Position
